# A novel cannula with a movable tip facilitates cannulation during endoscopic retrograde cholangiopancreatography in reconstructed intestinal tracts

**DOI:** 10.1055/a-2068-8606

**Published:** 2023-05-04

**Authors:** Tsuyoshi Suda, Satoko Inagaki, Saiho Sugimoto, Kiichiro Kaji, Shuichi Terasaki

**Affiliations:** Department of Gastroenterology, Kanazawa Red Cross Hospital, Kanazawa, Ishikawa, Japan

An 83-year-old woman was admitted with obstructive jaundice. She was diagnosed with pancreatic head cancer after a close examination ( Fig. 1 ). She had previously undergone total gastrectomy with Roux-en-Y reconstruction and caudal pancreatectomy for pancreatic body cancer. Thus, we attempted to treat the obstructive jaundice via endoscopic retrograde cholangiopancreatography (ERCP) using a short-type single-balloon enteroscope.

**Fig. 1:**
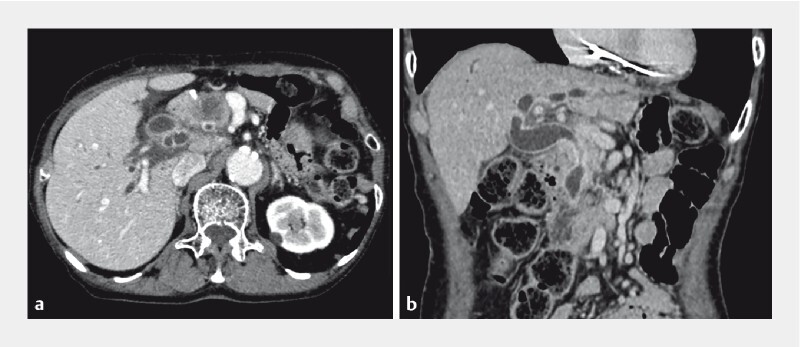
Obstructive jaundice due to pancreatic head cancer in a patient who previously underwent total gastrectomy with Roux-en-Y reconstruction.

The bile duct axis was not aligned due to the anatomic reconstruction, making intubation difficult and time consuming. The patient underwent placement of a covered self-expandable metal stent (cSEMS) followed by chemotherapy. However, ERCP was required again due to cSEMS deviation. Therefore, we planned to use a novel cannula with a movable tip (Zeon Medical Inc., Tokyo, Japan). The special feature of this cannula is that the area about 15 mm from the tip can be bent in two directions ( Fig. 2 , Video 1 ).

**Fig. 2:**
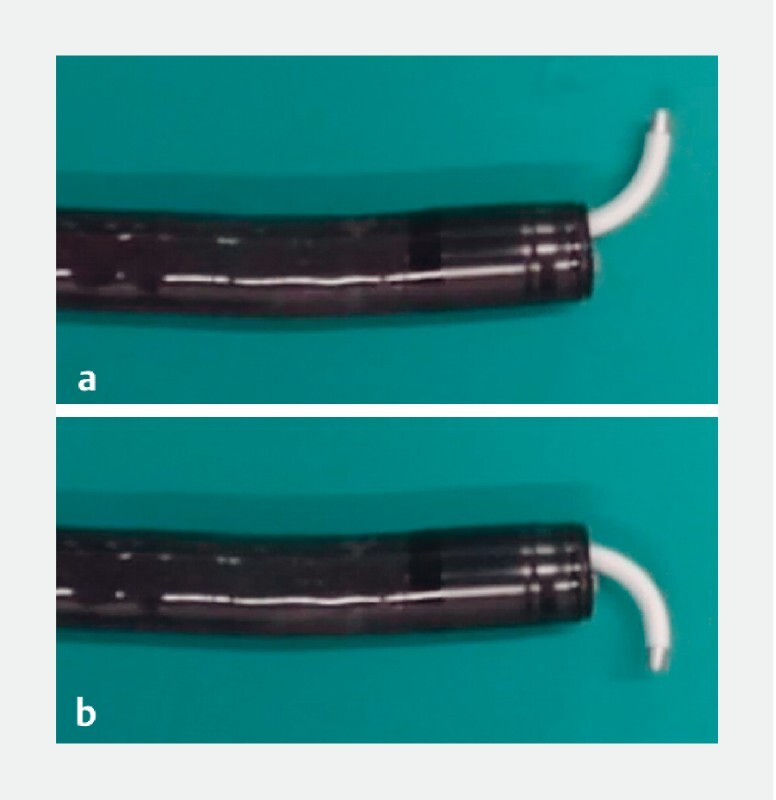
A novel cannula that bends in two directions near the tip.

**Video 1** A novel cannula that aligns with the bile duct axis by the bi-directional movement of the cannula tip. This cannula simplifies bile duct cannulation.


During ERCP ( Video 1 ), a regular cannula failed to intubate the bile duct because it could not align with the bile duct axis ( Fig. 3 ). The cannula with a movable tip could be turned around in the bile duct by moving the tip ( Fig. 4 ), capturing the bile duct axis, and successfully intubating the bile duct ( Fig. 5 ). It was then possible to place a plastic stent.

**Fig. 3:**
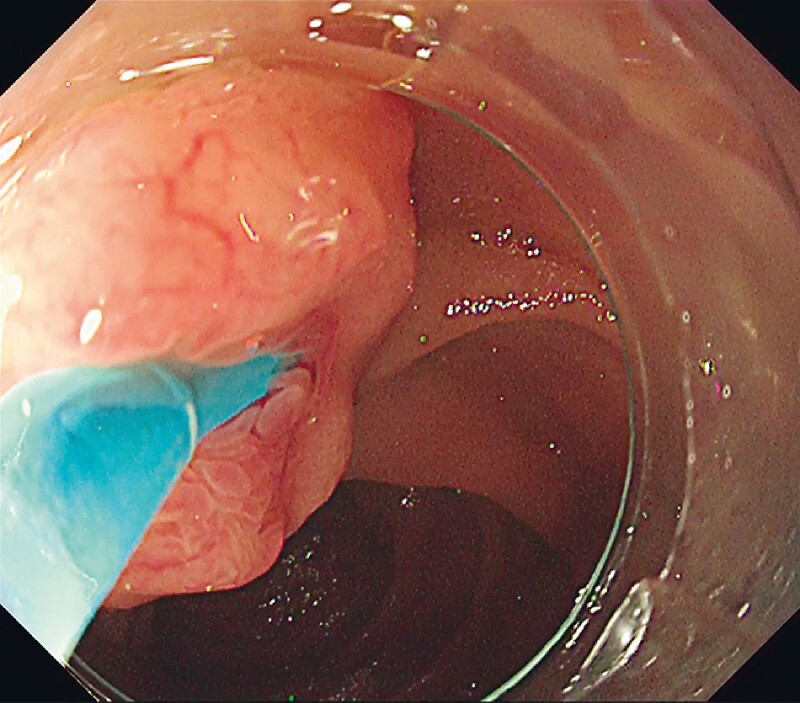
A regular cannula failed to intubate the bile duct.

**Fig. 4:**
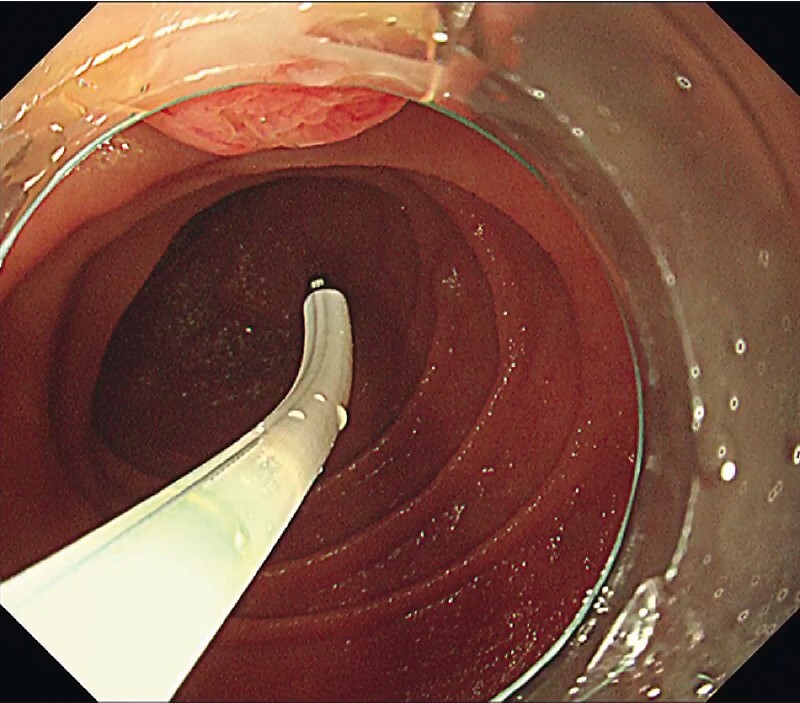
The tip can bend and move around in this novel cannula.

**Fig. 5:**
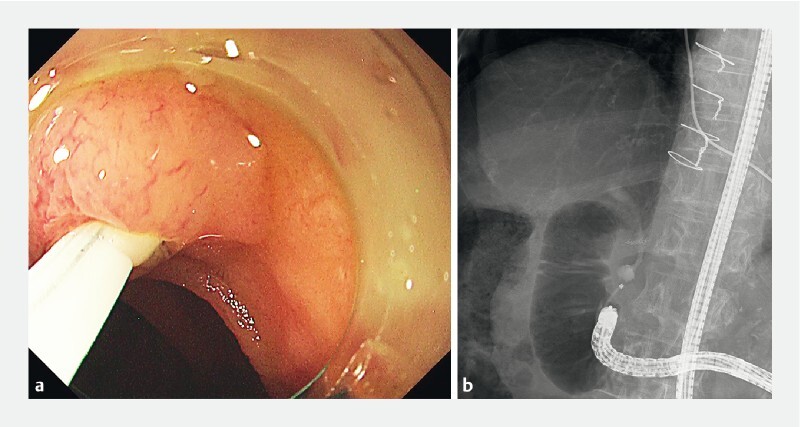
The bile duct was intubated successfully.

An endoscopic approach using a balloon endoscope is currently available for treating biliopancreatic diseases in reconstructed anatomy. There are also reports of ERCP using balloon-assisted enteroscopy 1 . However, many case reports describe difficulty in bile duct intubation due to reconstructed anatomy. In such cases, several solutions have been suggested during normal ERCP 2 . We also have previously reported a new method that involves a traction device in the case of reconstructed intestinal tract 3 . We consider that the movability of the tip of this novel cannula facilitates the alignment of the cannula with the bile duct axis. This approach can achieve bile duct intubation during ERCP in reconstructed intestinal tracts.

Endoscopy_UCTN_Code_TTT_1AR_2AC

## Notes

Endoscopy E-Videos

https://eref.thieme.de/e-videos
*E-Videos* is an open access online section of the journal *Endoscopy* , reporting on interesting cases and new techniques in gastroenterological endoscopy. All papers include a high-quality video and are published with a Creative Commons CC-BY license. Endoscopy E-Videos qualify for HINARI discounts and waivers and eligibility is automatically checked during the submission process. We grant 100% waivers to articles whose corresponding authors are based in Group A countries and 50% waivers to those who are based in Group B countries as classified by Research4Life (see: https://www.research4life.org/access/eligibility/ ). This section has its own submission website at https://mc.manuscriptcentral.com/e-videos
